# *GhNHX3D*, a Vacuolar-Localized Na^+^/H^+^ Antiporter, Positively Regulates Salt Response in Upland Cotton

**DOI:** 10.3390/ijms22084047

**Published:** 2021-04-14

**Authors:** Junping Feng, Wenyu Ma, Zongbin Ma, Zhongying Ren, Yang Zhou, Junjie Zhao, Wei Li, Wei Liu

**Affiliations:** 1 Collaborative Innovation Center of Henan Grain Crops, Agronomy College, Henan Agricultural University, Zhengzhou 450002, China; 18838916993@163.com (J.F.); zongbinma@henau.edu.cn (Z.M.); 2 Weinan Vocational and Technical College, Weinan 714026, China; 82101172178@caas.cn; 3 State Key Laboratory of Cotton Biology, Institute of Cotton Research of the Chinese Academy of Agricultural Sciences, Anyang 455000, China; renzhongying@caas.cn (Z.R.); zhaojunjie@caas.cn (J.Z.); liwei@caas.cn (W.L.); 4 Hainan Key Laboratory for Biotechnology of Salt Tolerant Crops, College of Horticulture, Hainan University, Haikou 570228, China; zhouyang@hainanu.edu.cn

**Keywords:** Na^+^/H^+^ antiporter, *Gossypium hirsutum*, vacuolar, ion content, salt tolerance

## Abstract

Vacuolar sodium/proton (Na^+^/H^+^) antiporters (NHXs) can stabilize ion contents to improve the salt tolerance of plants. Here, *GhNHX3D* was cloned and characterized from upland cotton (*Gossypium hirsutum*). Phylogenetic and sequence analyses showed that *GhNHX3D* belongs to the vacuolar-type NHXs. The GhNHX3D-enhanced green fluorescent protein (eGFP) fusion protein localized on the vacuolar membrane when transiently expressed in *Arabidopsis* protoplasts. The quantitative real-time PCR (qRT-PCR) analysis showed that *GhNHX3D* was induced rapidly in response to salt stress in cotton leaves, and its transcript levels increased with the aggravation of salt stress. The introduction of *GhNHX3D* into the salt-sensitive yeast mutant ATX3 improved its salt tolerance. Furthermore, silencing of *GhNHX3D* in cotton plants by virus-induced gene silencing (VIGS) increased the Na^+^ levels in the leaves, stems, and roots and decreased the K^+^ content in the roots, leading to greater salt sensitivity. Our results indicate that *GhNHX3D* is a member of the vacuolar NHX family and can confer salt tolerance by adjusting the steady-state balance of cellular Na^+^ and K^+^ ions.

## 1. Introduction

Plant sodium/proton (Na^+^/H^+^) antiporters (NHXs) are membrane-localized proteins widely existing in higher plants, which play an essential regulatory role in maintaining intracellular pH and ion balance [1,2]. Researchers first detected a Na^+^/H^+^ antiporter in the roots of barley (*Hordeum vulgare*) [3] and later discovered the transport activity of NHX in the storage tissues of the roots of beet (*Beta vulgaris*) [4]. The first gene encoding a vacuolar membrane Na^+^/H^+^ antiporter to be cloned in higher plants was *AtNHX1* from *Arabidopsis thaliana* [5]. Because NHXs play an important role in the salt tolerance of plants, many more Na^+^/H^+^ antiporters have been identified from other plants, including *Oryza sativa* [6], *Atriplex gmelini* [7], *Beta vulgaris* [8], and *Glycine max* [9]. In *A. thaliana*, six members of the Na^+^/H^+^ antiporter (*AtNHX1–6*) have been characterized and can be classified into Class I (*AtNHX1–4*) and Class II (*AtNHX5–6*) [10]. Studies have shown that Class I NHXs are located on the vacuolar membrane and have the same affinity for Na^+^ and K^+^, which can separate the Na^+^ and K^+^ accumulated in the cytoplasm into the vacuolar membrane to maintain cell turgor [11,12]. Class II NHXs are located on the endosomal membrane and mainly regulate the balance of K^+^, which respond to salt stress by accumulating K^+^ in the cell [13]. There are some conserved regions in the structure of vacuolar Na^+^/H^+^ antiporters isolated from different organisms. Plant vacuolar NHX protein usually contains about 530 amino acids, and its typical topological structure consists of about 9–12 transmembrane regions at the N-terminal and a hydrophilic C-terminal [7,14,15,16,17]. The transmembrane domain is sensitive to amiloride, a competitive inhibitor of Na^+^, and its derivatives, and is responsible for the transport function of the protein [17]. The C-terminal hydrophilic region is located on the vacuolar membrane and regulates the transport activity of the NHX protein [18].

The role and underlying mechanism of genes encoding Na^+^/H^+^ antiporters in response to salt stress have been thoroughly analyzed in many plant species. *AgNHX1* in *A. gmelini* and *AtNHX1* in *A. thaliana* show lower transcript levels under normal conditions, but their transcript levels increase with increasing or prolonged salt stress [7,10]. The vacuolar NHX proteins from barley roots and sunflower roots have no NHX transport activity under non-stress conditions, but their transporter activity is induced under salt stress [19,20]. Thus, plants can increase salt resistance by inducing the expression of Na^+^/H^+^ antiporters or activating existing inactive Na^+^/H^+^ antiporters under salt stress.

The vacuolar-localized NHX is a popular protein in the field of Na^+^/H^+^ antiporter research. Previous studies have shown that the insertion of genes encoding vacuolar NHXs into plants can effectively improve their salt tolerance. Transgenic *A. thaliana* plants over-expressing *AtNHX1* could grow under 200 mmol/L NaCl. Compared with the wild type, transgenic *A. thaliana* plants showed increased Na^+^ contents and stronger Na^+^/H^+^ antiporter activity [21]. The heterologous expression of *AtNHX1* in plants such as *Ipomoea batatas* [22], *Fagopyrum esculentum* [23], and *Festuca arundinacea* [24] led to increased intracellular Na^+^ concentrations, a higher K^+^/Na^+^ ratio, and improved tolerance to salt stress. The overexpression of *BnNHX1* also improved the salt tolerance of tobacco plants [25]. Therefore, it is presumed that plant vacuolar Na^+^/H^+^ antiporters can specifically recognize and compartmentalize Na^+^, thereby increasing the salt tolerance of plants. However, in-depth analyses of vacuolar Na^+^/H^+^ antiporters have suggested that their salt tolerance mechanism may involve the mediation of K^+^ accumulation and the maintenance of a low Na^+^/K^+^ ratio. In previous studies, tomato overexpressing *AtNHX1* could partition more K^+^ into the vacuole and cause feedback inhibition of Na^+^ absorption by increasing the content of K^+^ in the vacuole, thereby improving the salt tolerance of the plant [26]. Transgenic rice plants overexpressing *PgNHX1* could survive and complete their life cycle, setting flowers and seeds successfully. Whereas the upper leaves of wild type had a higher Na^+^ content, those of the transgenic lines had a higher K^+^ content [27]. Heterologous expression of *TNHXS1-IRES-TVP1* in transgenic tobacco caused the leaves to accumulate less Na^+^ and more K^+^ [28]. The results of those studies suggest that the mechanisms by which vacuolar-type NHX proteins regulate salt tolerance may differ among different plants.

Recent studies have shown that upland cotton vacuolar NHX can improve the salt tolerance of plants. Salt stress was found to strongly induce the accumulation of *GhNHX1* transcripts in cotton seedlings. The overexpression of *GhNHX1* in transgenic tobacco allowed them to grow naturally under 200 mM NaCl [29]. Functional analysis of *GhNHX1A/D* using virus-induced gene silencing (VIGS) proved that *GhNHX1A/D* positively affects the cotton response to salt stress [30]. Further experiments are required to clarify the mechanism by which this vacuolar NHX protein confers salt tolerance in cotton.

In preliminary work in our laboratory, we identified NHX family members from the whole genome of upland cotton and analyzed the transcript levels of their encoding genes under salt stress [31]. The most strongly induced member of the NHX gene family was the vacuolar Na^+^/H^+^ antiporter gene *GhNHX3D*. However, its salt tolerance mechanism and role in plant resistance to salt stress were unclear. In this study, *GhNHX3D* was cloned from the upland cotton cv. TM-1, and the GhNHX3D-enhanced green fluorescent protein (eGFP) fusion protein was used to clarify its subcellular localization. Then, a salt-sensitive yeast deletion mutant and a cotton *TRV:GhNHX3D*-silenced strain were used as materials to explore its role in salt tolerance. The Na^+^ and K^+^ contents were detected in the leaves, stems, and roots of the *TRV:GhNHX3D*-silenced cotton lines. The results confirm the important role of *GhNHX3D* in maintaining an appropriate Na^+^/K^+^ ratio in plant organs under salt stress and lay the foundation for further understanding the salt resistance mechanism in cotton.

## 2. Results

### 2.1. Phylogenetic Analysis and Conserved Domains of GhNHX3D

To analyze the evolutionary relationship between *GhNHX3D* and other plants Na^+^/H^+^ antiporters, a phylogenetic tree was constructed based on the amino acid sequences of Na^+^/H^+^ antiporters from *G. hirsutum*, *A. thaliana*, *Populus euphratica*, *O. sativa*, and *Zea mays*. As shown in the phylogenetic tree (Figure S1), the 28 NHX proteins from five species were clustered into two classes, namely the vacuolar-type and the endosomal-type. The vacuolar-type NHX group contained 20 *NHX* genes, and the endosomal-type contained eight *NHX* genes. *GhNHX3D* was closely related to members of the vacuolar *NHX* family, such as *PeNHX4* and *AtNHX4*, but relatively distantly related to the endosomal *NHX* family members from *A. thaliana*, *P. euphratica*, *O. sativa*, *Z. mays*, and *G. hirsutum*. These results indicate that *GhNHX3D* is a member of the vacuolar-type NHX family and may have similar Na^+/^H^+^ antiporter activity.

**Figure 1:**
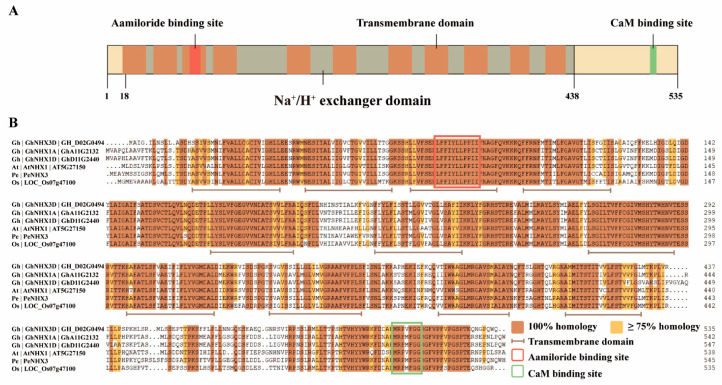
Protein sequence analysis of *GhNHX3D*. (**A**) Protein domain structure of *GhNHX3D* and (**B**) amino acid sequence alignment of *GhNHX3D* with vacuolar NHX proteins from other plants. The brown mark represents the predicted transmembrane domain, the red mark represents the amiloride binding site, the green mark represents CaM binding site, and the gray mark represents the Na^+^/H^+^ exchanger domain. Gh: *Gossypium hirsutum*; At: *Arabidopsis thaliana*; Pe: *Populus euphratica*; Os: *Oryza sativa*.

The protein of *GhNHX3D* contained the Na^+^/H^+^ exchanger domain, which is the essential structural domain of Na^+^/H^+^ transports, and had 11 conserved transmembrane regions (Figure 1A). When the amino acid sequence of *GhNHX3D* was compared with those of other plants vacuolar NHXs, it showed high homology with other plants vacuolar-type NHX proteins (Figure 1B). All vacuolar-type NHXs had a binding site of amiloride (a competitive inhibitor of Na^+^) in the third transmembrane domain and a conserved CaM binding site at the C-terminal.

### 2.2. Subcellular Localization Analysis of GhNHX3D

**Figure 2:**
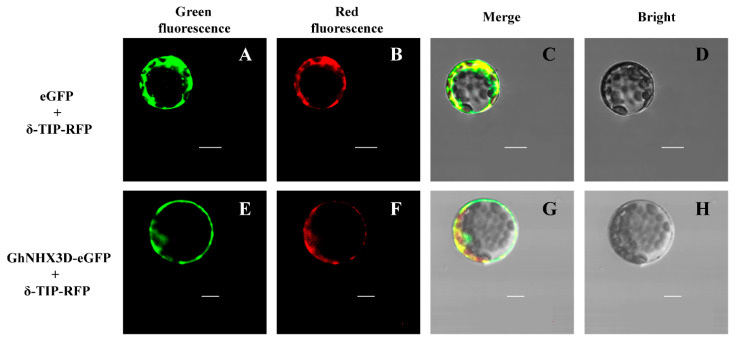
Subcellular localization of GhNHX3D-eGFP in *Arabidopsis* protoplasts. *GhNHX3D* fused with eGFP (**A**–**D**) and δ-TIP fused with red fluorescent protein (RFP) (**E**–**H**) were transiently expressed in *Arabidopsis* protoplast cells. δ-TIP protein is a localization marker for the vacuolar membrane. Bar = 10 μm.

Bioinformatics analyses showed that the *GhNHX3D* protein was closely related to vacuolar-type NHXs and had a conserved sequence of vacuolar Na^+^/H^+^ antiporters. These findings suggested that *GhNHX3D* may be located on the vacuolar membrane. We fused eGFP to *GhNHX3D*, and then the vacuolar marker δ-TIP-RFP and GhNHX3D-eGFP were co-introduced into *Arabidopsis* protoplasts for transient expression. As observed by laser confocal microscopy, in *Arabidopsis* protoplasts harboring eGFP and δ-TIP-RFP vectors, the green fluorescence produced by eGFP covered the entire cytoplasm, and the red fluorescence produced by δ-TIP-RFP was distributed on the vacuolar membrane (Figure 2A–D). When the fusion protein of δ-TIP-RFP and GhNHX3D-eGFP was expressed in *Arabidopsis* protoplasts, the yellow fluorescence produced by the combination of green and red fluorescence was located on the vacuolar membrane (Figure 2E–H), confirming that *GhNHX3D* localized on the vacuolar membrane.

### 2.3. Expression Profiles of GhNHX3D under Different Salinity Treatments

**Figure 3:**
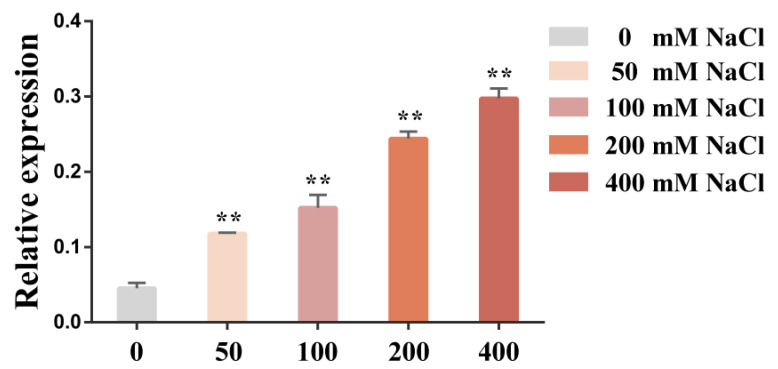
Relative transcript levels of *GhNHX3D* in cotton seedlings under salt stress. The salt concentration is shown on the x-axis; relative gene transcript levels are shown on the y-axis. Data are means ± standard deviation of three biological replicates. Significant differences compared with the 0 mM NaCl treatment (Student’s *t*-test): ** *p* < 0.01.

In our previous study, we showed that under NaCl stress, the transcript level of *GhNHX3D* in the leaves gradually increased and peaked at 6 h but decreased as the treatment time was extended to 12 h [31]. To further analyze the effect of salt treatments on the expression of *GhNHX3D* in upland cotton leaves, 4-week-old cotton seedlings were treated with 0, 50, 100, 200, and 400 mM NaCl for 6 h. As the NaCl concentration increased, the transcript level of *GhNHX3D* in the leaves showed an increasing trend (Figure 3). Thus, the expression of *GhNHX3D* was induced and regulated by salt stress.

### 2.4. Expression of GhNHX3D in Transgenic Yeast Cells

**Figure 4:**
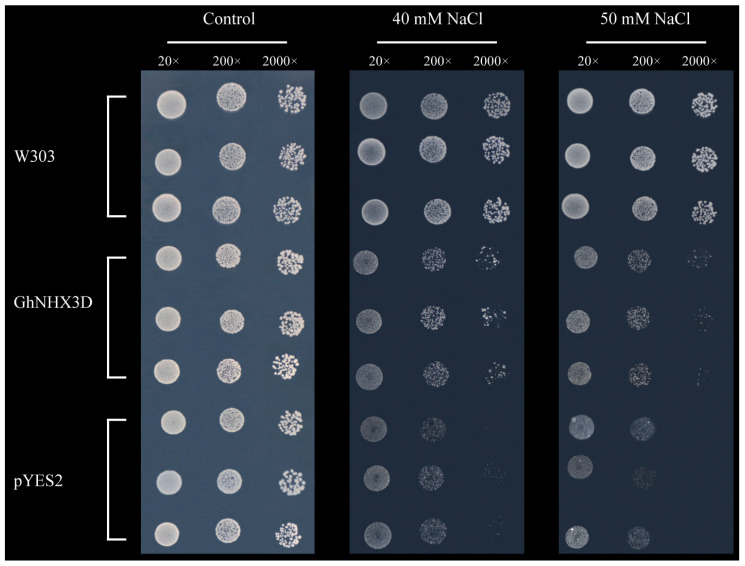
Expression of *GhNHX3D* in yeast mutants. *GhNHX3D*: yeast mutant strain AXT3 transformed with plasmid pYES2-*GhNHX3D*; pYES2: yeast mutant strain AXT3 transformed with the empty plasmid pYES2; W303: wild-type yeast strain W303. 20×, 200×, and 2000× represent dilutions.

The results of the salt tolerance analysis of transgenic yeast cells expressing *GhNHX3D* are shown in Figure 4. In the medium without NaCl, the wild-type yeast W303, yeast harboring the empty vector pYES2, and yeast harboring *GhNHX3D* were able to grow naturally. In the medium containing 40 mM NaCl, W303 was still able to naturally when diluted 2000-times, while the yeast cells harboring pYES2 were inhibited and could not grow. The growth of the strain harboring pYES2-*GhNHX3D* was similar to that of W303, and its growth was inhibited slightly after being diluted 2000-times.

When the NaCl concentration was increased to 50 mM, the growth of W303 was not significantly inhibited, while the growth of pYES2 and pYES2-*GhNHX3D* harboring cells were inhibited to varying degrees. Compared with pYES2, pYES2-*GhNHX3D* grew better. After 200-times and 2000-times dilution, there was a small amount of growth, while pYES2 hardly grew after 200-times dilution. These experimental results indicate that pYES2-*GhNHX3D* complemented the salt-sensitive characteristic of the yeast mutant.

### 2.5. GhNHX3D Positively Regulates Salinity Resistance in Cotton

**Figure 5:**
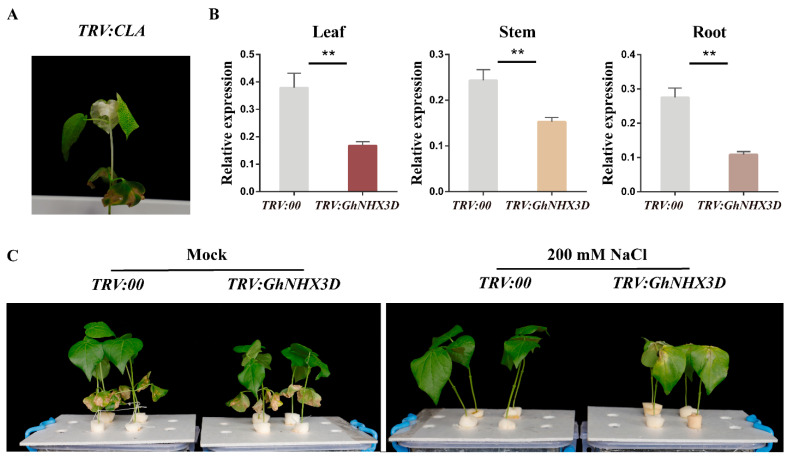
Silencing of *GhNHX3D* in upland cotton. (**A**) *TRV:CLA* is an albino phenotype used as a positive control. (**B**) *GhNHX3D* transcript levels in *TRV:00* and TRV:*GhNHX3D*. *GhHIS3* was used as an internal control. Data are means ± standard deviation of three biological replicates. Significant differences between groups (Student’s *t*-test): ** *p* < 0.01. (**C**) Phenotypes of *TRV:00* and *TRV:GhNHX3D* under mock and 200 mM NaCl treatment for 10 days.

Next, we used the VIGS approach to silence *GhNHX3D* in cotton seedlings to investigate its potential roles in salt stress resistance, according to the previous study [32]. The *TRV:GhNHX3D* interference vector was constructed. The constructs *TRV:00*, *TRV:CLA*, and *TRV:GhNHX3D* were each infiltrated into the back of cotyledons of 10-day-old cotton seedlings. After 12 days (when the leaves of the positive control plant showed an albino phenotype), RNA was extracted from the leaves, stems, and roots of the cotton plants to analyze gene transcript levels (Figure 5A). As shown in Figure 5B, the transcript levels of *GhNHX3D* in leaves, stems, and roots were significantly lower in the silenced line than in the empty vector control. To ensure the specificity of the silencing effect, the transcript levels of *GhNHX3A*, a homologous gene of *GhNHX3D*, were also tested. The transcript levels of *GhNHX3A* in the leaves, stems, and roots did not differ significantly between *TRV:GhNHX3D* and *TRV:00* plants (Figure S2). Thus, *GhNHX3D* was efficiently and specifically silenced in the *TRV:GhNHX3D* cotton plants.

After the silencing efficiency was confirmed, VIGS plants with the strongest silencing of *GhNHX3D* were selected for salt treatment. The *TRV:GhNHX3D* and *TRV:00* plants were treated with 0 mM (mock) or 200 mM NaCl for 10 days, and their salt tolerance was determined (Figure 5C). The *GhNHX3D*-silenced plants were more sensitive to salt than the control plants.

**Figure 6:**
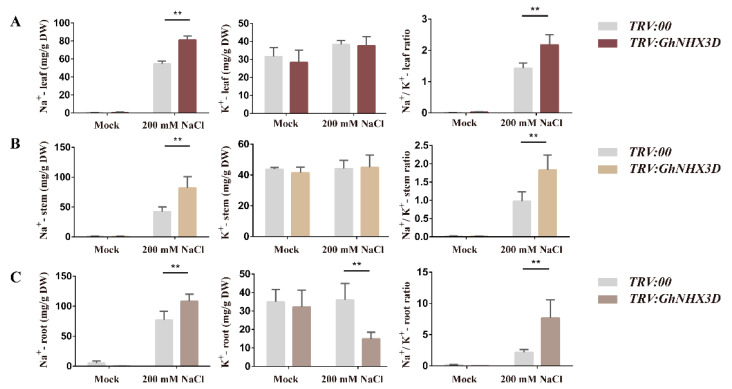
Changes in Na^+^ and K^+^ contents and Na^+^/K^+^ ratio in organs of *TRV:GhNHX3D* and *TRV:00* cotton plants under salt stress. (**A**) leaves; (**B**) stems; (**C**) roots. Data are means ± standard deviation of three biological replicates. Significant differences between groups (Student’s *t*-test): ** *p* < 0.01.

The salt tolerance of plants is related to the ion contents in the cells. Excessively high ion concentrations in the cell are toxic, resulting in reduced salt tolerance [33,34]. In this study, we used atomic absorption spectrophotometry (AAS) to measure the Na^+^ and K^+^ contents in the leaves, stems, and roots of *TRV:GhNHX3D* and *TRV:00* plants to further explore the salt tolerance mechanism of *GhNHX3D* (Figure 6). The results showed that in the control group (no NaCl), the contents of Na^+^ and K^+^, and the ratio of Na^+^/K^+^ in the leaves, stems, and roots of *TRV:GhNHX3D* and *TRV:.000* plants were approximately the same. After treatment with 200 mM NaCl, the Na^+^ content in the leaves, stems, and roots was significantly higher in *TRV:GhNHX3D* plants than in *TRV:00* plants, and the K^+^ content in the roots was significantly lower in *TRV:GhNHX3D* plants than in *TRV:00* plants. The K^+^ content in the stems and leaves was approximately the same in *TRV:GhNHX3D* and *TRV:00* plants.

The accumulation of Na^+^ in the leaves, stems, and roots of *GhNHX3D*-silenced plants increased significantly under salt stress, alongside a decrease in the K^+^ content in the roots, which led to a continuous increase in the Na^+^/K^+^ ratio in *GhNHX3D*-silenced plants. Under salt stress, compared with *TRV:00* plants, the *GhNHX3D*-silenced plants showed a significantly higher Na^+^/K^+^ ratio in the leaves, stems, and roots. Thus, compared with *TRV:00* plants, *TRV:GhNHX3D* plants accumulated more Na^+^ in their organs under salinity stress, and the roots had a lower K^+^ content. This resulted in a higher Na^+^/K^+^ ratio, which increased sensitivity to salinity stress.

## 3. Discussion

### 3.1. GhNHX3D Is a Vacuolar-Localized Na^+^/H^+^ Antiporter

Plant Na^+^/H^+^ antiporters can be divided into two types, the vacuolar-type, and the endosomal-type, according to their different locations [10,13]. In our analyses, *GhNHX3D* showed higher homology to vacuolar NHXs than to endosomal ones (Figure S1). Prediction of the conserved domains of *GhNHX3D* showed that it contains the Na^+^/H^+^ exchanger domain that is the typical functional domain of NHX proteins (Figure 1A). The analysis of the transmembrane region of the amino acid sequence of *GhNHX3D* showed that it contains 11 transmembrane domains (Figure 1B), which is similar to AtNHX1 [5]. The third transmembrane region contains a binding site of amiloride, as found in NHXs from *Suaeda salsa* [15], *A. thaliana* [5], and *O. sativa* [6]. Studies have confirmed that 5-(N-methyl-N-isobutyl)-amiloride (MIA) can significantly inhibit NHX transport activity in rice roots [35]. We speculated that the transport activity of *GhNHX3D* from upland cotton might be similarly inhibited by MIA. *GhNHX3D* has a conserved CaM binding site, which is known to be essential for transport activity and ion selectivity [17,18]. A region comprising eight amino acids (S/T,P/F,G,X,S/T,Ø,X,V) is important for targeting vacuolar Na^+^/H^+^ antiporters to the vacuolar membrane [36]. The ninth transmembrane region of *GhNHX3D* contains eight amino acids (Figure S3). These findings provide further evidence that *GhNHX3D* is a vacuolar Na^+^/H^+^ antiporter.

To determine the subcellular localization of *GhNHX3D*, the vacuolar membrane marker constructs δ-TIF-RFP and GhNHX3D-eGFP were co-expressed in *Arabidopsis* protoplasts (Figure 2). Microscopic observations confirmed that *GhNHX3D* is localized on the vacuolar membrane, consistent with the localization of HtNHX1 [36] and GhNHX1A/D [30]. The results of the evolutionary protein structure and subcellular localization analyses indicate that *GhNHX3D* was located on the vacuolar membrane and belongs to the vacuolar NHX family. Therefore, we speculated that *GhNHX3D* has a function similar to that of vacuolar Na^+^/H^+^ antiporters and may be involved in the maintenance of the vacuolar Na^+^/K^+^ balance and other physiological processes.

### 3.2. GhNHX3D Can Confer the Salt Tolerance of Gossypium hirsutum

In this study, the results of quantitative real-time PCR (qRT-PCR) showed that the transcript level of *GhNHX3D* increased at 6 h after exposure to NaCl stress at different concentrations (Figure 3), indicating that it responds positively during the early stage of salt stress in upland cotton. Moreover, the expression of *GhNHX3D* was significantly induced by 400 mM NaCl than other lower concentrations, similar to the upregulation of *SsNHX1*, *HtNHX1*, and *HtNHX2* under NaCl stress [15,36], indicating that *GhNHX3D* may have similar functions to that of these genes and also plays an important role in the salt tolerance process.

Previous studies have shown that the salt sensitivity of yeast mutants can be partially compensated by the heterologous expression of plant vacuolar transporter genes. Therefore, the functional complementation in yeast mutants can be used to explore the function of plant Na^+^/H^+^ antiporters [37]. In previous studies, *AtNHX1* and *AtNHX2* were able to improve the salt tolerance of yeast mutants [5,10]. Yeast mutants expressing the vacuolar Na^+^/H^+^ antiporter *AgNHX1* showed partial restoration of the ability to survive in a high salt environment [7]. The vacuolar Na^+^/H^+^ antiporter *HvNHX1* increased the tolerance to Na^+^ of yeast mutants to an extent similar to the wild type [38]. In the present study, the expression of *GhNHX3D* in the yeast Na^+^/H^+^ antiporter mutant ATX3 allowed it to resume growth under a high NaCl concentration (Figure 4). This result demonstrates that *GhNHX3D* could compensate for the lack of the vacuolar Na^+^/H^+^ antiporter in yeast and play an essential role in establishing salt tolerance.

Many studies have shown that Na^+^/H^+^ antiporters can effectively improve the resistance of plants to salt stress and regulate the ion balance in cells [39,40,41]. In this study, the expression of *GhNHX3D* was efficiently and specifically reduced using VIGS (Figure 5B). Compared with cotton plants harboring the empty vector (*TRV:00*), the *TRV:GhNHX3D* cotton plants treated with 200 mM NaCl were more sensitive to salt with withered leaves and inhibited growth (Figure 5C).

The results of previous studies have shown that vacuolar NHXs can partition Na^+^ into vacuoles in plants under salt stress, thereby maintaining the ion balance in the cell [39,42]. Further studies on Na^+^/H^+^ antiporters have shown that vacuolar NHXs have the same affinity for K^+^/H^+^ as Na^+^/H^+^ and can simultaneously catalyze the exchange of Na^+^/H^+^ and K^+^/H^+^ [43]. Overexpression of *AtNHX1* in tomatoes resulted in higher vacuolar K^+^/H^+^ reversal activity [26]. Transgenic alfalfa (*Medicago sativa* L.) expressing *TaNHX2* accumulated more K^+^ in leaves than did wild-type plants [44]. Compared with wild type, transgenic rice plants expressing *PgNHX1* showed better growth and higher K^+^ and lower Na^+^ contents in their leaves [27]. The results of those studies show that different NHX proteins have different transport modes for Na^+^ and K^+^.

We measured the Na^+^ and K^+^ contents in the experimental plants by AAS (Figure 6). After salt stress, compared with *TRV:00* plants, *TRV:GhNHX3D* plants showed significantly increased Na^+^ contents in the leaves and stems, but similar K^+^ contents. In the roots of the *TRV:GhNHX3D* plants, the Na^+^ content increased significantly, and the K^+^ content decreased significantly. This led to a continuous increase in the Na^+^/K^+^ ratio in the silenced plants, which reduced their resistance to salt stress. Previous studies propose a model that the vacuolar NHX protein mainly exerts a protective function through the vacuolar separation of Na^+^ and K^+^ to minimize salt stress, thereby preventing the Na^+^/K^+^ ratio from becoming toxic in the cytosol [45]. Therefore, whether the vacuolar NHX protein enhances the salt tolerance of plants through Na^+^ compartmentation depends on the selectivity of the vacuolar NHX protein under different salt stress environments and the ionic environment in the cytoplasm. The results of the present study indicate that *GhNHX3D* may mediate K^+^ compartmentation in the roots of cotton and maintain low levels of Na^+^ in the leaves, stems, and roots to regulate the steady-state balance of Na^+^ and K^+^ ions in the cell, thereby improving the salt tolerance of cotton.

## 4. Materials and Methods

### 4.1. Plant Materials and Treatments

The tops of the seed husk of upland cotton cv. TM-1 seeds were removed with scissors to expose the tip of the radicle. The cut cotton seeds were placed in sterile water in a 30 °C incubator to germinate overnight. Seeds with undamaged radicles of the same length were selected and rinsed with water. Seed shells were removed with tweezers, and the seed was sown with the radicle pointing downwards in vermiculite. The seeds were covered with a layer of mulch to retain moisture and then transferred to a light incubator (23 °C and 60% humidity with a 16 h light/8 h dark photoperiod). After 2–3 days, the seed tops had emerged from the soil layer. The mulch film and seed husk were gently removed, and then the seedlings were placed in a hydroponic tank containing Hoagland’s nutrient solution. Cotton seedlings that were strong and consistent in size were selected for treatment with 0, 50, 100, 200, and 400 mM NaCl. The second true leaves from three seedlings per treatment were harvested after 6 h of treatments and stored at −80 °C. Three biological replicates were performed for each treatment.

### 4.2. Bioinformatics Analysis

To analyze the evolutionary relationship between *GhNHX3D* and NHX proteins from other species, we collected the NHX protein sequences of *G. hirsutum*, *A. thaliana*, *P. euphratica*, *O. sativa*, and *Z. mays* (Table S1) [46,47]. Then, MEGA X was used for multiple sequence alignment, and a phylogenetic tree was constructed using the neighbor-joining method with 1000 bootstrap replications. DNAMAN 9.0 was used to perform multiple sequence alignments of *GhNHX3D* and vacuolar NHXs from other species (Table S1). DOG 2.0 software was used to display the protein conserved domains of *GhNHX3D*. The transmembrane region of *GhNHX3D* was predicted and analyzed using tools at the TMHMM Server v. 2.0 (http://www.cbs.dtu.dk/services/TMHMM/ accessed on 17 February 2021).

### 4.3. Cloning of GhNHX3D

The leaves of *G. hirsutum* (cv. TM-1) frozen at −80 °C were crushed into a powder in liquid nitrogen with a mortar and pestle, and then total RNA was extracted using TRIzol reagent (TIANGEN, Beijing, China). A NanoDrop 2000 instrument (Thermo Fisher Scientific, Waltham, Massachusetts, USA) was used to measure the concentration and purity of RNA, and agarose gel electrophoresis was used to detect the integrity of RNA. The cDNA was synthesized by reverse transcription in vitro using the PrimeScript™ II 1st Strand cDNA Synthesis Kit (TaKaRa, Dalian, China), using 1 μg total RNA as the template.

The *GhNHX3D* (*GH_D02G0494*) sequence was downloaded from the Cotton Functional Genomics Database (http://www.cottonfgd.org/ accessed on 17 February 2021). Then, the primers GhNHX3D-1F/ GhNHX3D-1R (Table S2) were designed according to the sequence. The *GhNHX3D* sequence was amplified using the primers with the cDNA obtained by reverse transcription as the template and Tks Gflex DNA Polymerase (TaKaRa, Dalian, China). The reaction mixture was 50 μL. The thermal cycling conditions were as follows: 94 °C pre-denaturation 1 min; 98 °C denaturation 40 s, 55 °C annealing 30 s, and 68 °C extension for 50 s over 30 cycles; final extension at 68 °C for 8 min. The amplified product was detected by 1.2% agarose gel electrophoresis. The target gene fragment was purified and connected with the T vector. The ligated product was transformed into *Escherichia coli* competent cells, and the positive clones were selected for sequence verification after being cultured at 37 °C.

### 4.4. Quantitative Real-Time PCR Analysis

The leaves of cotton plants under salt stress were quickly frozen in liquid nitrogen, and then total RNA was extracted using TRIzol reagent (TIANGEN, Beijing, China). First-strand cDNA was synthesized from 1 μg total RNA with the PrimeScript™ RT reagent Kit with gDNA Eraser (Perfect Real Time) (TaKaRa, Dalian, China). The cDNA from leaves of plants treated with different salt concentrations was used as the template for qRT-PCR. The primers are shown in Table S2. The thermal cycling conditions were as follows: 95 °C pre-denaturation for 30 s; 95 °C denaturation for 10 s, 60 °C annealing for 30 s, and extension at 72 °C for 10 s over 40 cycles. Three technical replicates were performed per sample.

### 4.5. Subcellular Localization Analysis

The primers GhNHX3D-E-1F/GhNHX3D-E-1R (Table S2) were designed to amplify the full-length open reading frame of *GhNHX3D* (without the stop codon). The product was digested with BamHI/EcoRI and then connected to the pCAMBIA2300 (35S-eGFP) vector to obtain GhNHX3D-eGFP.

In an ultra-clean workbench, leaves were cut into thin strips with a sterile blade; then, the leaf slices were placed in 10 mL mixed enzymolysis solution from the Arabidopsis Protoplast Preparation and Transformation Kit (Coolaber, Beijing, China). The leaf slices were shaken in this solution at 40 rpm at room temperature in the dark for about 3 h until most of the leaf tissue was transparent. The undigested leaf tissue was removed from the solution, and the container and undigested leaf slices were washed three times with 10 mL II-W5 solution. The solution and washing liquid were combined in a 50 mL centrifuge tube and centrifuged at 100× *g* for 2 min. The supernatant was removed, and then 5 mL pre-chilled II-W5 solution was added. The protoplasts in the pellet were gently resuspended using a pipette. The mixture was then centrifuged at 100× *g* for 1 min, and the supernatant was discarded. The protoplasts were resuspended in 5 mL pre-chilled II-W5 solution and then incubated on ice for 30 min. The mixture was centrifuged at 100× *g* for 1 min, the supernatant was removed, and the protoplasts were resuspended in a corresponding volume of MMg solution to a final density of 2 × 10^5^/mL.

Purified plasmid (10–20 µg) was added to a centrifuge tube, and then 100 µL protoplasts were added. After mixing gently, 110 µL IV-PEG solution was added. The mixture was gently combined and left at room temperature for 15 min before adding 0.44 mL II-W5 solution to halt the transformation process. The mixture was centrifuged at 100× *g* at room temperature for 1 min, and then the supernatant was removed. The cells were resuspended in 1 mL II-W5 solution. The centrifuge tube was placed horizontally in a light incubator at 23 °C, avoiding strong light, and incubated for 12–20 h. Finally, the transformed cells were observed under a laser scanning confocal microscope (FV 1200, Olympus, Japan).

### 4.6. Functional Assays of GhNHX3D in a Yeast Mutant

The pYES2 empty vector and pYES2-*GhNHX3D* were transformed into the yeast mutant strain AXT3 (ena1−4Δ: HIS3, nha1Δ:LEU2, nhx1Δ:TRP1) by the LiAc method [48,49], and positive clones of transformed yeast were obtained by screening. The yeast wild-type W303 (MAT: ura3-1, leu2-3, 112 his3-11, 15 trp1-1, ade2-1, and can1-100) and the strains harboring pYES2 and pYES2-*GhNHX3D* were cultured to a saturated state, then 10 μL of culture solution was diluted 20-, 200-, and 2000- times successively. An 8 μL aliquot of each of the diluted bacterial solutions was added to APG medium (10 mM arginine, 8 mM phosphoric acid, 2% galactose, 2 mM MgSO_4_, 1 mM KCl, 0.2 mM CaCl_2_, and trace minerals and vitamins, adjusted to pH to 5.6 with arginine) containing 0, 40, or 50 mM NaCl and cultured at 30 °C for about 5 days before photographing growth.

### 4.7. Virus-Induced Gene Silencing in Cotton Plants

The silencing fragment of *GhNHX3D* was amplified and inserted into the VIGS vector (*TRV:00*) to generate the *GhNHX3D*-silencing construct. Details of the primers used are listed in Table S2. *TRV1* was mixed in equal amounts with *TRV:CLA*, *TRV:00*, and *TRV:GhNHX3D*, respectively. Then, each mixture was infiltrated into the cotyledon of 10-day-old cotton seedlings using a syringe. When the albino phenotype occurred on the *TRV:CLA* cotton seedlings, the *TRV:00* and *TRV:GhNHX3D* cotton seedlings were collected for silencing efficiency and specificity analysis by qRT-PCR and subjected to NaCl treatment. After 10 days of the salt treatment, the second true leaves, stems, and roots of the *TRV:00* and *TRV:GhNHX3D* plants under the salt treatment and mock were collected for measurement of Na^+^ and K^+^ contents. At least 30 cotton seedlings were used for each experimental group, and the assays were conducted with three biological replicates.

### 4.8. Determination of Total Na^+^ and K^+^ Contents in Cotton Organs

The second true leaves, stems, and roots of the *TRV:00* and *TRV:GhNHX3D* plants were dried at 90 °C, and then ground to a powder. Next, 0.05 g ground sample was dissolved in 5 mL concentrated HNO_3_ (i.e., nitrification). The solution was diluted with deionized water 12 times and then centrifuged. The supernatant was collected to analyze the ion content by AAS.

## 5. Conclusions

We successfully cloned *GhNHX3D* encoding an Na^+^/H^+^ antiporter from upland cotton. Bioinformatics analyses show that *GhNHX3D* is closely related to vacuolar-type Na^+^/H^+^ antiporters. A subcellular localization analysis revealed that *GhNHX3D* is located on the vacuolar membrane. Therefore, *GhNHX3D* is presumed to be a member of the vacuolar NHX family. qRT-PCR analysis revealed that *GhNHX3D* transcripts increased in cotton leaves along with the NaCl concentration increased. The introduction of *GhNHX3D* into a salt-sensitive yeast mutant improved its salt tolerance, confirming that its encoded protein plays a role in salt tolerance. Cotton plants with silenced *GhNHX3D* showed increased sensitivity to salt. Analysis of the Na^+^ and K^+^ contents in different tissues indicated that *GhNHX3D* affects salt tolerance by adjusting the balance of Na^+^ and K^+^ in roots and by regulating the Na^+^ content in leaves and stems to maintain a low Na^+^/K^+^ ratio under salt stress.

## Data Availability

The data presented in this study are available in this article and supplementary material.

## Supplementary Materials

The following are available online at https://www.mdpi.com/article/10.3390/ijms22084047/s1. Table S1. Protein sequences used in this study. Table S2. Primers used in this study. Figure S1. Phylogenetic analysis of *GhNHX3D* protein. Figure S2. The expression of *GhNHX3A* in the *TRV:00* and *TRV:GhNHX3D* plants. Figure S3. The conserved eight amino acids for targeting to the vacuolar membrane.
