# Supplementary figures and images for: GhNHX3D, a Vacuolar-Localized Na+/H+ Antiporter, Positively Regulates Salt Response in Upland Cotton
Source: Int J Mol Sci. 2021 Apr 14;22(8):4047. doi: 10.3390/ijms22084047 (PMC8070948; doi:10.3390/ijms22084047)

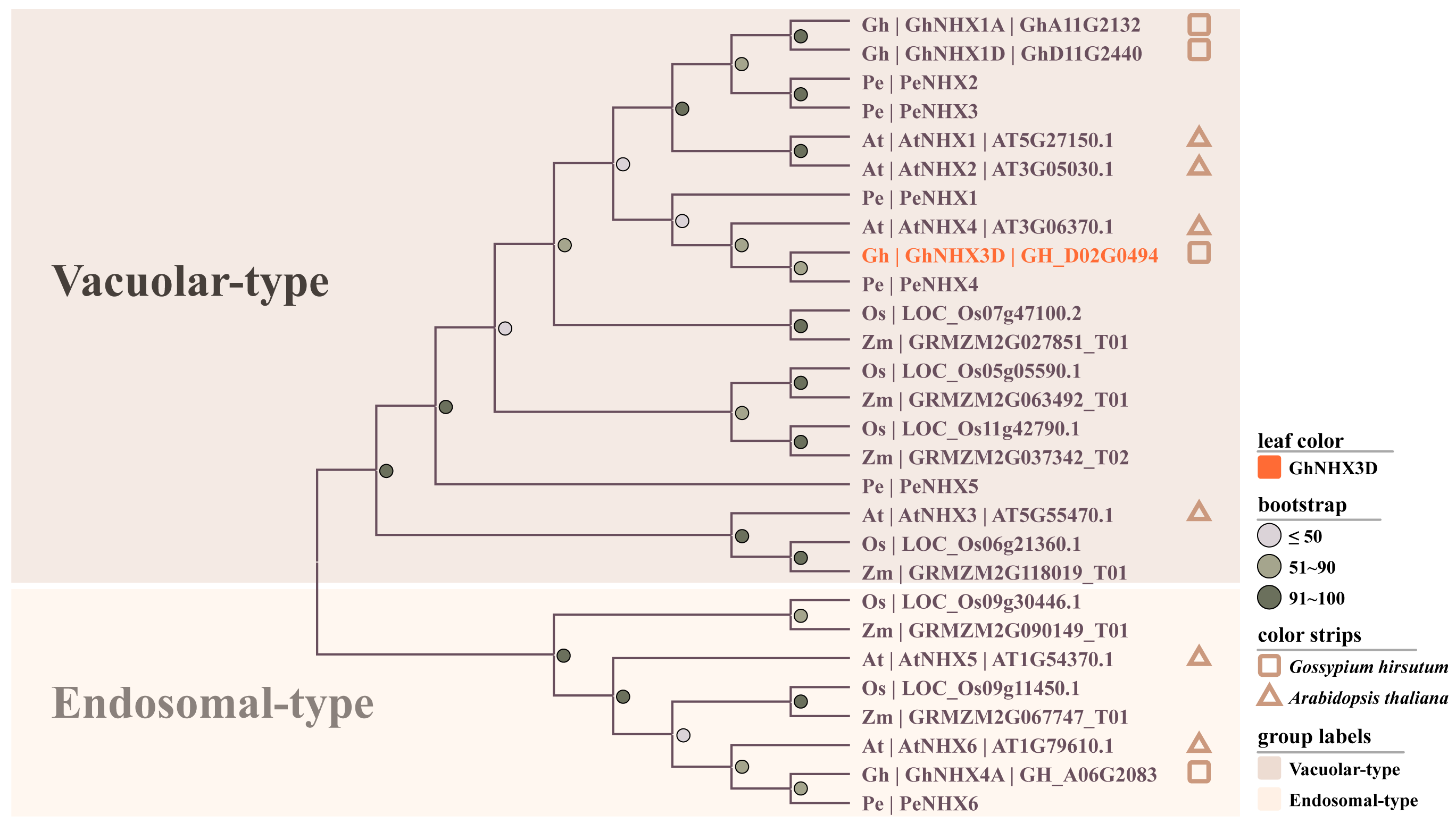

Supplement: Supplementary file 1 [file ijms-22-04047-s001.zip › Supplementary Files/Figure S1.tif]

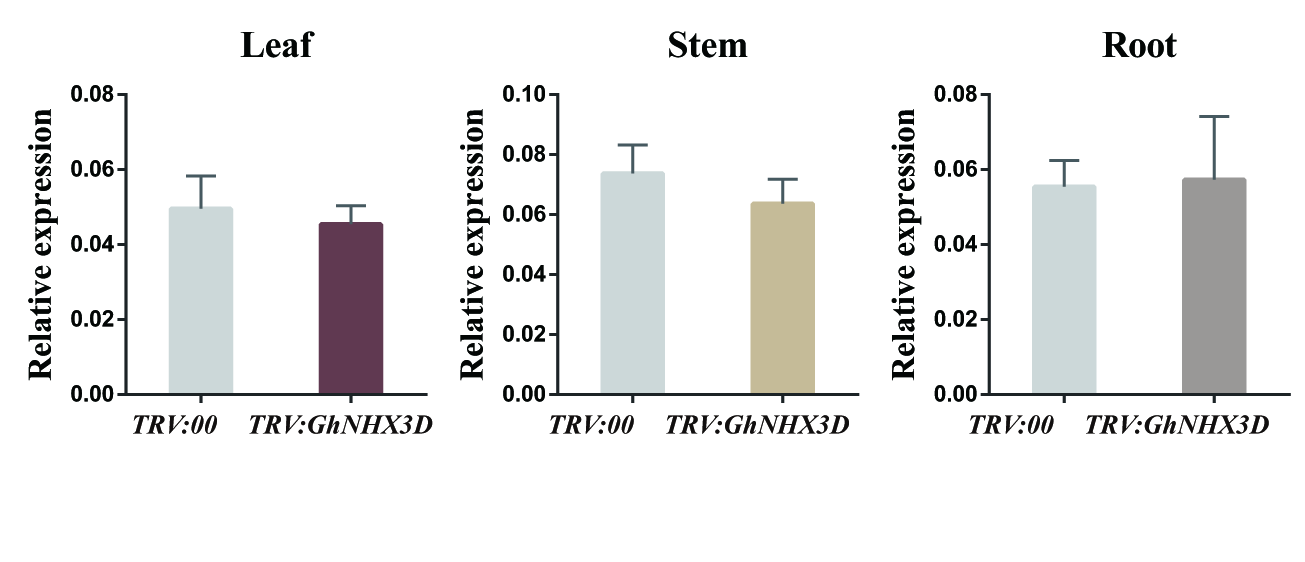

Supplement: Supplementary file 1 [file ijms-22-04047-s001.zip › Supplementary Files/Figure S2.tif]

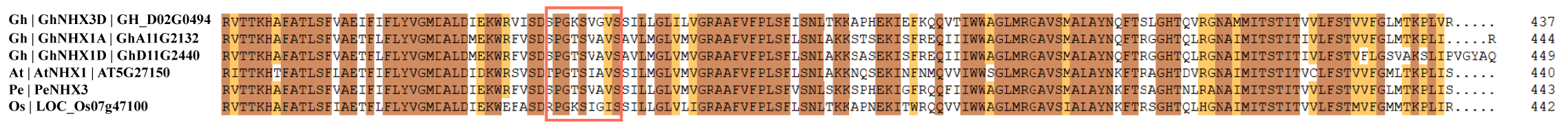

Supplement: Supplementary file 1 [file ijms-22-04047-s001.zip › Supplementary Files/Figure S3.tif]
